# Local Aspects of Avian Non-REM and REM Sleep

**DOI:** 10.3389/fnins.2019.00567

**Published:** 2019-06-05

**Authors:** Niels C. Rattenborg, Jacqueline van der Meij, Gabriël J. L. Beckers, John A. Lesku

**Affiliations:** ^1^Avian Sleep Group, Max Planck Institute for Ornithology, Seewiesen, Germany; ^2^Cognitive Neurobiology and Helmholtz Institute, Utrecht University, Utrecht, Netherlands; ^3^School of Life Sciences, La Trobe University, Melbourne, VIC, Australia

**Keywords:** sleep, bird, mammal, unihemispheric, atonia, evolution, slow wave, propagation

## Abstract

Birds exhibit two types of sleep that are in many respects similar to mammalian rapid eye movement (REM) and non-REM (NREM) sleep. As in mammals, several aspects of avian sleep can occur in a local manner within the brain. Electrophysiological evidence of NREM sleep occurring more deeply in one hemisphere, or only in one hemisphere – the latter being a phenomenon most pronounced in dolphins – was actually first described in birds. Such asymmetric or unihemispheric NREM sleep occurs with one eye open, enabling birds to visually monitor their environment for predators. Frigatebirds primarily engage in this form of sleep in flight, perhaps to avoid collisions with other birds. In addition to interhemispheric differences in NREM sleep intensity, the intensity of NREM sleep is homeostatically regulated in a local, use-depended manner within each hemisphere. Furthermore, the intensity and temporo-spatial distribution of NREM sleep-related slow waves varies across layers of the avian hyperpallium – a primary visual area – with the slow waves occurring first in, and propagating through and outward from, thalamic input layers. Slow waves also have the greatest amplitude in these layers. Although most research has focused on NREM sleep, there are also local aspects to avian REM sleep. REM sleep-related reductions in skeletal muscle tone appear largely restricted to muscles involved in maintaining head posture. Other local aspects of sleep manifest as a mixture of features of NREM and REM sleep occurring simultaneously in different parts of the neuroaxis. Like monotreme mammals, ostriches often exhibit brainstem-mediated features of REM sleep (muscle atonia and REMs) while the hyperpallium shows EEG slow waves typical of NREM sleep. Finally, although mice show slow waves in thalamic input layers of primary sensory cortices during REM sleep, this is not the case in the hyperpallium of pigeons, suggesting that this phenomenon is not a universal feature of REM sleep. Collectively, the local aspects of sleep described in birds and mammals reveal that wakefulness, NREM sleep, and REM sleep are not always discrete states.

## Introduction

Wakefulness and sleep, as well as the two types of sleep found in mammals and birds – rapid eye movement (REM) and non-REM (NREM) sleep – are often treated as mutually exclusive states distinguished by a suite of behavioral, electroencephalographic, and electromyographic traits. In mammals and birds, NREM sleep is distinguished from wakefulness by the presence of high-amplitude slow waves in electroencephalogram (EEG) or local field potential (LFP) recordings. These slow waves result from the slow oscillation, or alternation, of neuronal membrane potentials between a hyperpolarized state with neuronal quiescence and a depolarized state with action potentials (Steriade et al., 1993; Reiner et al., 2001; Steriade, 2006). Slow waves – typically quantified as slow wave activity (SWA) (approximately 0.5–4.5 Hz power density) – increase and decrease as a function of time spent awake and asleep, respectively, in both mammals and birds, suggesting that they reflect homeostatically regulated processes (Jones et al., 2008; Martinez-Gonzalez et al., 2008; Rattenborg et al., 2009; Lesku et al., 2011b). However, although thalamocortical spindles are present during NREM sleep in mammals (Astori et al., 2013), they are apparently absent in birds (van der Meij et al., 2019a). EEG activity during wakefulness and REM sleep are similar in both mammals and birds; although the hippocampal theta rhythm present in mammals has not been detected in birds (Rattenborg et al., 2011). REM sleep is distinguished from wakefulness by increased arousal thresholds and reduced muscle tone (only partial in birds; Dewasmes et al., 1985), intermittently interrupted by twitching of skeletal muscle groups, including those controlling eye movements. As in mammals, thermoregulatory responses are suppressed during avian REM sleep (Heller et al., 1983; Graf et al., 1987; Scriba et al., 2013), and in both altricial mammals and birds, the amount of REM sleep is highest in young, decreasing across early ontogeny (Roffwarg et al., 1966; Jouvet-Mounier et al., 1970; Scriba et al., 2013). Finally, REM sleep is homeostatically regulated in both groups (Tobler and Borbély, 1988; Martinez-Gonzalez et al., 2008; Newman et al., 2008; Lesku et al., 2011b).

To explore the mechanisms and functions of NREM and REM sleep, researchers examine changes in the time spent in each state following experimental manipulations. Interspecific differences in the time spent in these states is also used to identify biological or ecological traits that predict their variation, and thereby, possibly, the functions of each state (Lesku et al., 2009). Quantifying the time spent in each state necessarily requires treating wakefulness, NREM sleep, and REM sleep as mutually exclusive states. However, in practice, the transitions from one state to another can be gradual (e.g., wakefulness to NREM sleep), rendering the boundary between states arbitrary. In some species, sleep and wakefulness can also occur unihemispherically, a specialized adaptation that enables animals to partially sleep under ecological circumstances when being fully asleep would be disadvantageous (Rattenborg et al., 2000, 2016; Lyamin et al., 2008b, 2018). However, such *local sleep* is not restricted to these animals. As new technology allows more brain regions to be examined simultaneously, it is becoming evident that even in conventional mammalian models for studying sleep, such as rodents and humans, traits used to define different states can occur at the same time in different brain regions (Nir et al., 2011, 2017; Vyazovskiy et al., 2011b; Emrick et al., 2016; Funk et al., 2016; Tamaki et al., 2016; Durán et al., 2018). Herein, we review the various types of local sleep found in birds, including those not known to occur in mammals.

## Local Aspects of NREM Sleep

### Asymmetric and Unihemispheric Sleep

The discovery that some animals can engage in NREM sleep with one cerebral hemisphere at a time (unihemispheric sleep), or more deeply with one hemisphere than the other (asymmetric sleep) undoubtedly contributed to current views regarding the local nature of NREM sleep (Krueger and Obál, 1993; Lima and Rattenborg, 2007; Rattenborg et al., 2012; Siclari and Tononi, 2017; Castelnovo et al., 2018; Krueger et al., 2019). These asymmetric forms of NREM sleep (also known as unihemispheric or asymmetric slow wave sleep) have been described in Odontocete cetaceans (e.g., dolphins, porpoises, and the beluga whale) (Mukhametov et al., 1977; Lyamin et al., 2008b), pinnipeds in the superfamily Otarioidea (fur seals, sea lions, and walruses) (Lyamin et al., 2012, 2018), one manatee (order Sirenia) (Mukhametov et al., 1992), and several species of birds (Spooner, 1964; Peters et al., 1965; Ookawa and Takagi, 1968; Ball et al., 1988; Szymczak et al., 1996; Rattenborg et al., 1999a,b, 2000, 2001, 2016; Ayala-Guerrero et al., 2003; Ookawa, 2004; Low et al., 2008; Fuchs et al., 2009). In the mammalian and avian species in which eye state was examined in conjunction with EEG recordings, usually the eye contralateral to the sleeping (or more deeply sleeping) hemisphere is closed while the eye contralateral to the awake (or more lightly sleeping) hemisphere is open (Peters et al., 1965; Ookawa and Takagi, 1968; Ball et al., 1988; Rattenborg et al., 1999a,b, 2001; Lyamin et al., 2002, 2004; Low et al., 2008; Fuchs et al., 2009).

#### A Note on Nomenclature

Although asymmetric forms of NREM sleep occur in marine mammals and birds, comparing the extent of this phenomenon across taxonomic groups has been hindered by the use of different approaches to characterize these forms of sleep. The criteria for classifying NREM sleep as unihemispheric, asymmetric, or symmetric (bihemispheric) varies across studies depending, in part, on the relative emphasis given to EEG or behavioral indicators of sleep. In studies that emphasize EEG activity, the boundaries between these states are defined by the degree of interhemispheric asymmetry in NREM sleep-related EEG SWA. In marine mammals, an asymmetry index (AI) in the level of SWA has been calculated as: AI = (left hemisphere SWA – right SWA)/(left SWA + right SWA) (Lyamin et al., 2008a,b). The commonly used thresholds, AI ≤-0.3 or ≥0.3 and ≤-0.6 or ≥0.6, reflect increasing degrees of interhemispheric asymmetry, with the former usually considered asymmetric and the later unihemispheric; however, the exact term (i.e., asymmetric or unihemispheric) ascribed to episodes of mammalian NREM sleep exceeding these thresholds varies across studies (Lyamin et al., 2008a,b, 2016, 2018). Historically, in birds, the relationship between unilateral eye closure and interhemispheric asymmetries in EEG slow waves was simply mentioned anecdotally (Spooner, 1964; Peters et al., 1965; Ookawa and Takagi, 1968). Later, Ball et al. (1988) showed that visually identified periods of pronounced interhemispheric asymmetry in EEG slow waves were usually associated with unilateral eye closure in glaucous-winged gulls (*Larus glaucescens*). Later, Rattenborg et al. (1999a,b) showed that unilateral eye closure was associated with a statistically significant average interhemispheric asymmetry in SWA in mallard ducks (*Anas platyrhynchos*), with SWA levels in the hemisphere contralateral to the open eye higher than (bihemispheric) wakefulness, but lower than that occurring during bihemispheric NREM sleep (Rattenborg et al., 1999a,b; see also Fuchs et al., 2009). Given that the mallards had one eye open and seemed to use this eye to monitor their environment (see below), such asymmetries were classified as unihemispheric NREM sleep. Based on the relationships between unilateral eye closure and interhemispheric asymmetries in SWA described in EEG studies, eye state alone has been used as an indicator of unihemispheric sleep in several behavioral studies (Ball et al., 1988; Mascetti and Vallortigara, 2001; Boerema et al., 2003; Nelini et al., 2010, 2012; Mascetti, 2016). Finally, the AI thresholds used in dolphins and seals were recently employed to classify NREM sleep as unihemispheric, asymmetric, or symmetric in great frigatebirds (*Fregata minor*) in the wild where eye state could not be monitored (Rattenborg et al., 2016).

None of the terms used to describe the asymmetric forms of sleep and wakefulness fully capture all EEG and behavioral aspects of these states. Unihemispheric sleep inherently implies unihemispheric wakefulness. This seemingly makes sense when applied to fur seals, but not dolphins. When sleeping in the water, fur seals float on one side with their nostrils held above the surface to breath. This posture is maintained by unilateral movements of the front flipper in the water which is presumably controlled by the contralateral awake hemisphere. Interestingly, the eye facing down in the water is open while the other is closed (Lyamin et al., 2004), possibly enabling seals to detect predatory sharks and orcas; this might explain why fur seals in the wild float on their side during the day, when they could visually detect predators, but not at night (Trites et al., 2009). Consequently, in fur seals, the EEG asymmetry is clearly associated with a motor asymmetry, as well as an apparent sensory asymmetry associated with having one eye open. Hence, the term unihemispheric sleep (or unihemispheric wakefulness) accurately describes the neurophysiology and the behavior. Dolphins also have one eye open during unihemispheric sleep. However, unlike fur seals, dolphins can swim in a coordinated and directed manner while sleeping unihemispherically, indicating that at least some subcortical motor systems are bilaterally “awake” (Siegel, 2008). Consequently, the term unihemispheric sleep fails to adequately describe all waking aspects of this state in dolphins. Similarly, the converse, emphasizing the bilateral wake-like behavior and categorizing a dolphin’s state as fully awake when they are swimming with one eye closed and exhibiting unilateral cortical slow waves, obviously deemphasizes the behavioral (eye closure) and unilateral EEG signs of sleep (Siegel, 2008). Overall, given the mosaic of behavioral and EEG signs of wakefulness and sleep occurring in marine mammals and birds, it is perhaps not surprising that researchers have struggled to find simple terms that adequately describe these complex states.

#### Adaptive Use of the Open Eye

Determining whether sleeping with one eye open serves an adaptive function would likely enhance our understanding of asymmetric/unihemispheric sleep and the relative importance that should be given to eye state. One possibility is that having one eye open is simply a functionless byproduct of having one hemisphere awake (or sleeping less deeply) for some other reason. For example, when sleeping in the water, do fur seals keep one eye open to watch for threats, or is this simply an epiphenomenon of keeping one hemisphere awake to control unilateral flipper movements? Although it is unknown whether the open eye can detect threats in fur seals, research on birds and dolphins provide insight into the answer. Captive ducks sleeping on the ground at the edge of a group spend a greater proportion of NREM sleep with one eye open (rather than both eyes closed) when compared to ducks sleeping flanked on both sides by other ducks (Rattenborg et al., 1999a,b). Moreover, only ducks at the group edge show a preference for looking away from the other birds when sleeping with one eye open. Given that they were not exhibiting any asymmetrical motor activity, this directional preference in and of itself strongly suggests that ducks are able to visually detect threats when sleeping with one eye open. Indeed, ducks respond rapidly to a threatening visual stimulus presented to the open eye, indicating that at least some components of wakefulness are online even though the corresponding hemisphere shows SWA levels intermediate between unequivocal NREM sleep and unequivocal wakefulness. In the wild, this may be sufficient for detecting approaching predators. Nonetheless, further research is needed to identify the brain regions that mediate this anti-predator response and to determine whether other aspects of wakefulness are compromised by the presence of intermediate levels of SWA in the hemisphere connected to the open eye.

Dolphins also actively use the open eye to monitor their environment during unihemispheric sleep. However, in contrast to ducks, captive dolphins direct the open eye toward conspecifics. Pacific white-sided dolphins (*Lagenorhynchus obliquidens*) swimming side-by-side in a group of four directed their open eye toward the other dolphins (Goley, 1999). In addition, during the first few weeks following birth, bottlenose dolphin mothers and their calves keep the eye facing each other open and the other closed when sleeping while continuously swimming (Gnone et al., 2006; Sekiguchi et al., 2006; Lyamin et al., 2007). Importantly, in both species, the dolphins were swimming side-by-side in a circle around their pool. Consequently, despite exhibiting the same asymmetry in motor activity needed to swim in a given circular direction, the dolphins on the inside and outside of the circle kept a different eye open and different hemisphere awake. Although the responsiveness of the open eye has not been examined, the lack of a relationship with motor activity and the preference for directing the open eye toward conspecifics suggest that maintaining visual awareness is one function of unihemispheric sleep in dolphins. Finally, in addition to maintaining unilateral visual awareness, if the ear connected to the awake hemisphere remains responsive to sounds, unihemispheric sleep may explain how dolphins are able to sustain periods of acoustic responsiveness for several days (Ridgway et al., 2006, 2009; Branstetter et al., 2012).

#### Unihemispheric and Asymmetric Sleep in Flight

The research outlined above suggests that asymmetric forms of sleep instill animals with some of the adaptive benefits of wakefulness. However, research on sleep in flying birds suggests that these partial waking states are insufficient to meet all ecological demands for wakefulness, at least under certain circumstances. Several avian species engage in long, non-stop flights: bar-tailed godwits (*Limosa lapponica baueri*) fly from Alaska to New Zealand in 8.1 days spanning 11,690 km of sustained flight (Gill et al., 2009); great frigatebirds fly around the Indian Ocean for up to 2 months without landing on the water (Weimerskirch et al., 2016); and Alpine swifts (*Tachymarptis melba*) and Common swifts (*Apus apus*) can fly for 200 and 300 days, respectively (Liechti et al., 2013; Hedenström et al., 2016). Many other birds also make multiday, non-stop flights (reviewed in Rattenborg, 2017). The discovery that dolphins can swim in a coordinated manner during unihemispheric sleep and ducks can switch to asymmetric sleep when needed on the ground, led to the assumption that flying birds maintain aerodynamic control and navigation by sleeping with one eye open (Rattenborg, 2017).

**FIGURE 1:**
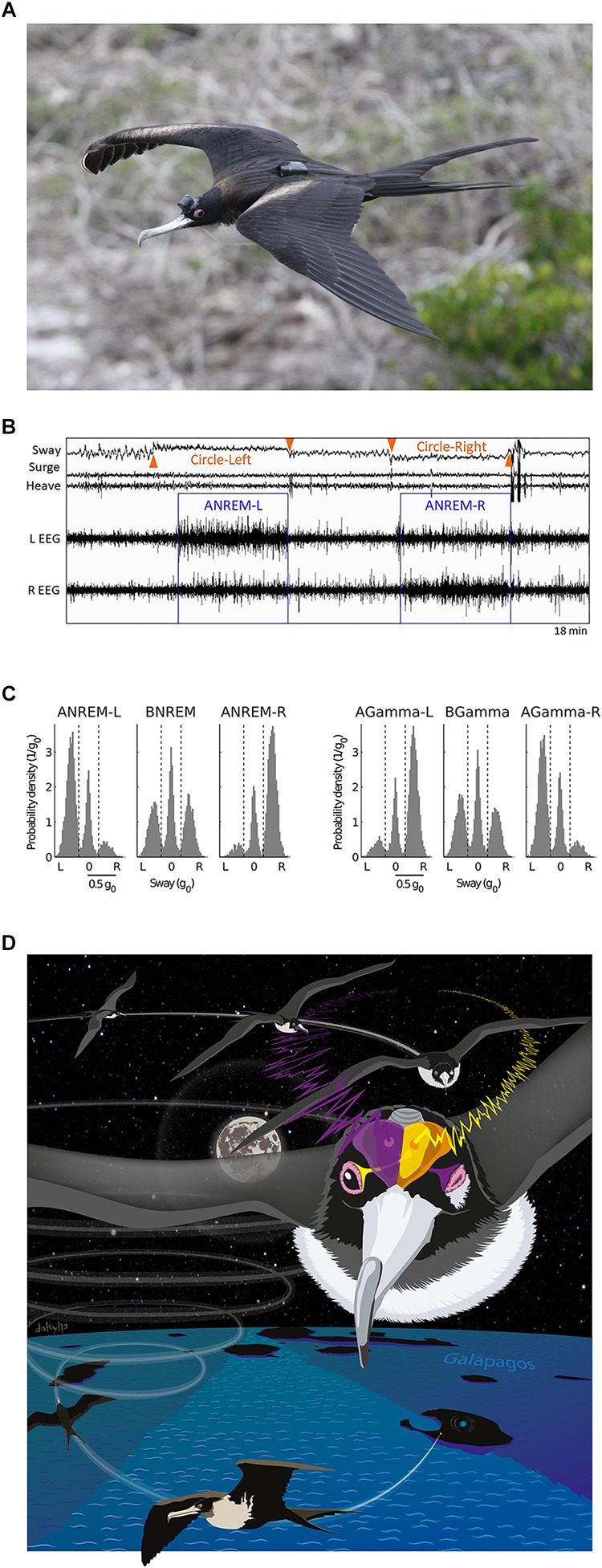
Sleep in flight. **(A)** Female great frigatebird with a head mounted data logger for recording the EEG from both cerebral hemispheres and triaxial acceleration. A GPS logger mounted on the back recorded position and altitude. **(B)** Electroencephalogram (EEG) and accelerometry (sway, surge, and heave) recording from a frigatebird sleeping while circling in rising air currents. When the bird circled to the left (as indicated by centripetal acceleration detected in the sway axis) the bird showed asymmetric NREM sleep (ANREM) with the left hemisphere sleeping deeper (larger slow waves) than the right (ANREM-L), and when the bird circled to the right the right hemisphere slept deeper than the left (ANREM-R); during the other recording segments the bird was awake. **(C)** The relationship between interhemispheric asymmetries in slow wave activity (SWA, 0.75–4.5 Hz) and gamma activity (30–80 Hz) during NREM sleep for all birds combined (*N* = 14). During ANREM, the birds usually circled toward the side with greater SWA and lower gamma activity. By contrast, during bihemispheric NREM (BNREM) without asymmetries in SWA or gamma (BGamma), the birds showed no preference for circling in one particular direction. AGamma-L and AGamma-R indicate NREM with greater gamma in the left and right hemispheres, respectively. **(D)** Illustration showing a bird circling to the right while sleeping with the right hemisphere. Although the birds’ eye state is not known, based on studies from other birds, the EEG asymmetries suggest that the frigatebirds kept the eye connected to the more awake (lower SWA and higher gamma) hemisphere open and facing the direction of the turn. Panels **(A–C)** reproduced with permission from Rattenborg et al. (2016). Photo by Bryson Voirin. Illustration by Damond Kyllo.

Sleep in flight was recently demonstrated for the first time in great frigatebirds (Rattenborg et al., 2016). Using a head-mounted EEG and acceleration data logger (Figure 1A), Rattenborg et al. (2016) found that sleep mainly occurred at night while the birds were soaring on rising air currents, and never when they flapped their wings. As expected, NREM sleep was more often asymmetric in flight when compared to sleep on land. In flight, nearly half of the asymmetric sleep exceeded the threshold for unihemispheric sleep (i.e., AI ≤-0.6 or ≥0.6), as typically defined in marine mammals. Episodes of asymmetric sleep were usually short, but could last up to several minutes (Figure 1B). Unexpectedly, the frigatebirds also engaged in episodes of bihemispheric sleep, which could also last for several minutes. However, this form of sleep did not affect their aerodynamic control of flight, raising the question, why did they usually sacrifice sleep in one hemisphere by primarily sleeping asymmetrically. The accelerometry recordings revealed when the birds were circling to the left or right, and this suggested that they relied on asymmetric sleep to watch where they were going. When circling, the hemisphere opposite to the direction of the turn showed the lowest SWA and the highest gamma (30–80 Hz) activity (Figure 1C), suggesting that the eye facing into the turn was open (Figure 1D). Given that frigatebirds have no predators when over the ocean, they might sleep this way to avoid head-on collisions with other birds circling in the opposite direction in the same air mass.

Nonetheless, other lines of evidence suggest that sleeping asymmetrically is not sufficient to meet all of their demands for wakefulness while on the wing at night. Despite spending most of the nighttime hours soaring, when sleep could occur, the frigatebirds slept <1 h per night. This suggests that even at night while soaring, when frigatebirds are not known to feed, their need for attention usually exceeds that afforded by sleeping with one hemisphere awake. Otherwise, one would have expected them to maximize the time spent sleeping asymmetrically or unihemispherically. Although the exact cognitive challenges that require bihemispheric wakefulness remain unclear, great frigatebirds follow eddies – productive parts of the ocean with increased foraging opportunities – at night, even though they only feed during the day (Tew Kai et al., 2009). By following eddies, frigatebirds may ensure that they are over a productive area at daybreak. How frigatebirds track eddies at night is unknown (Tew Kai et al., 2009), but the unexpectedly low amount of sleep in flight suggests that this and, perhaps, other cognitive demands require attention exceeding that occurring during asymmetric or unihemispheric sleep. Thus, although keeping an eye open may allow ducks, dolphins, fur seals, and, apparently, frigatebirds to visually monitor their environment during asymmetric or unihemispheric sleep, this unilateral awareness is probably not sufficient to meet all demands for wakefulness. Consequently, even in animals capable of asymmetric forms of sleep, the time spent sleeping may be greatly reduced under challenging ecological circumstances (Rattenborg et al., 2004, 2016; Lesku et al., 2012).

#### Unihemispheric or Asymmetric Sleep in Other Animals?

Collectively, these studies indicate that having one eye open to monitor the environment is an important adaptive component of asymmetric or unihemispheric sleep, even if processing in the contralateral hemisphere is limited. Interestingly, the ability to sleep with one eye open is common among birds (Ball et al., 1988), and might even predate their evolution (Rattenborg et al., 2000). Given that birds are reptiles in the taxonomic clade Dinosauria (Brusatte et al., 2015), non-avian reptiles might be expected to also sleep with one eye open. Indeed, non-avian reptiles, including crocodilians – the closest living relatives to birds – have been observed resting with one eye open (Rattenborg et al., 2000; Mathews et al., 2006; Kelly et al., 2015). Moreover, similar to ducks sleeping at the edge of a group, Western fence lizards (*Sceloporus occidentalis*) and juvenile saltwater crocodiles (*Crocodylus porosus*) direct the open eye toward potential threats (Mathews et al., 2006; Kelly et al., 2015). Despite these behavioral similarities, due to the lack of systematic electrophysiological studies, it is unclear whether closure of one eye is associated with asymmetric or unihemispheric sleep in non-avian reptiles (Rattenborg et al., 2000; Kelly et al., 2015).

The prevalence of sleep with one eye open in birds, and a potentially similar state in reptiles, questions why this type of sleep is not more widespread among mammals. Certainly, there are many terrestrial species that might benefit from being able to sleep asymmetrically or unihemispherically when needed. Interestingly, a recent study suggests that humans might be able to modulate the intensity of sleep unilaterally in response to ecologically relevant challenges (Tamaki et al., 2016). On the first night spent sleeping in a new (potentially dangerous) environment, people slept less deeply with parts of the left hemisphere (based on EEG SWA), whereas on the second night, both hemispheres slept deeply. Importantly, on the first, but not the second night, people were also more responsive to sounds presented to the right ear than the left, suggesting that the EEG asymmetry enhanced auditory awareness unilaterally. Given that this phenomenon was only recently discovered in humans, the capacity to modulate interhemispheric sleep intensity in response to changing ecological demands may be more widespread among terrestrial mammals than currently recognized. If so, it may have served as a precursor for the evolution of more asymmetric forms of sleep which evolved independently three times when the terrestrial ancestors to dolphins and porpoises, seals in the superfamily Otarioidea, and manatees transitioned to living and sleeping in a marine environment (Rattenborg et al., 2000).

### Local NREM Sleep Homeostasis

The discovery that NREM sleep can occur independently in the two hemispheres of some animals raised the question as to whether NREM sleep is homeostatically regulated independently in each hemisphere. Homeostasis can manifest as an increase in time spent in, or intensity of, NREM sleep, the latter measured as EEG SWA (Tobler, 2011). Early studies of dolphins did indeed suggest that NREM sleep is regulated intrahemispherically. Following unihemispheric sleep deprivation in dolphins, the hemisphere that had been kept awake usually (seven of nine experiments performed on five dolphins) showed an increase above preceding baseline levels in the time spent in NREM sleep (Oleksenko et al., 1992). Although the amount of recovery sleep did not correlate with the duration of sleep deprivation (see Lyamin et al., 2008b), it is possible that some of the recovery manifested as an increase in NREM sleep intensity. Unfortunately, SWA during NREM sleep was not quantified. In fur seals, the amount of bihemispheric NREM sleep on land increased following extended periods in the water, wherein sleep primarily occurred asymmetrically or unihemispherically (Lyamin et al., 2018). However, it is unclear whether the increase in bihemispheric sleep reflects two independent, intrahemispheric homeostatic responses occurring at the same time, a propensity for NREM sleep to primarily occur bihemispherically on land (Lyamin et al., 2008c), or both. As in the dolphin studies, SWA during NREM sleep was not reported. In future studies, it would be interesting to determine whether NREM sleep time or intensity increase unilaterally following sleep deprivation of only one hemisphere while fur seals sleep unihemispherically in the water. Finally, although unihemispheric sleep deprivation has not been performed in birds, several behavioral studies in chicken chicks have shown small, but significant, changes in the time spent with only the left or right eye closed in response to treatments thought to selectively activate one hemisphere or the other (e.g., Mascetti et al., 2007; Nelini et al., 2010, 2012; Quercia et al., 2018; reviewed in Mascetti, 2016), suggesting that sleep may be homeostatically regulated independently in each hemisphere. It will be important for future studies to determine the extent to which these changes in eye state correlate with changes in hemispheric sleep measured electrophysiologically (see Lesku et al., 2011b).

Even though the hemispheric regulation of NREM sleep needs further study in mammals and birds, several lines of evidence suggest the NREM sleep intensity is homeostatically regulated in a local, use-dependent manner *within* a hemisphere in both taxonomic groups (reviewed in Rattenborg et al., 2012). The local, use-dependent homeostatic regulation of NREM sleep-related SWA in the neocortex was established in a series of studies on humans (Kattler et al., 1994; Huber et al., 2004; Landsness et al., 2009) and rats (Vyazovskiy et al., 2000; Yasuda et al., 2005; Vyazovskiy and Tobler, 2008; Hanlon et al., 2009). In these studies, selectively activating certain cortical regions during wakefulness induced a local increase in EEG SWA in those regions during subsequent NREM sleep. Interestingly, reducing activation of a cortical region during wakefulness later diminished SWA in that region during sleep (Huber et al., 2006). Collectively, these studies demonstrate that the local level of NREM sleep intensity is determined by the prior local level of brain use, or disuse, during wakefulness in mammals.

**FIGURE 2:**
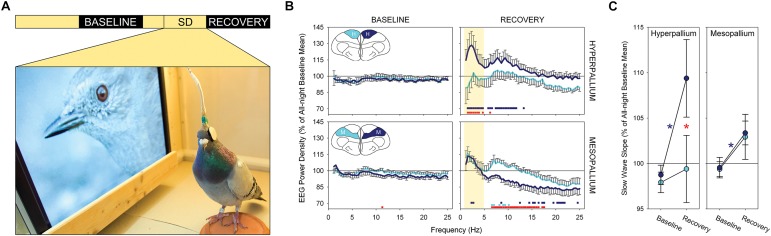
Local sleep homeostasis in the avian brain. **(A)** Experimental design: a 12 h baseline night, 8 h period of bihemispheric sleep deprivation with unilateral visual stimulation (SD) and a 12 h recovery night. Photograph shows the experimental environment during the treatment. **(B)** Spectral power density (0.78–25.00 Hz) during NREM sleep for the first quarter of the baseline and recovery nights for the stimulated (dark blue) and visually deprived (light blue) hyperpallia and mesopallia. Data are presented as mean ± SE. Colored squares at the bottom of each recovery night plot reflect a significant pairwise comparison between the baseline and recovery night of the stimulated (dark blue) and visually deprived (light blue) hyperpallia; red squares denote a significant asymmetry between the left and right brain region during recovery sleep. Although the experimental treatment induced interhemispheric asymmetries across a wide range of frequencies, slow wave activity (yellow shading) in the hyperpallium showed the largest asymmetry. Insets: frontal view of a transverse section through the cerebrum of a pigeon highlighting the hyperpallium (H) and mesopallium (M). **(C)** Up slope of NREM sleep slow waves in the hyperpallium and mesopallium contralateral to the stimulated eye (dark blue) and deprived eye (light blue) during the first quarter of the baseline and recovery night. Data are presented as mean ± SE. Significant changes in slope between the baseline and recovery nights are marked with an asterisk (contralateral to the stimulated eye in dark blue; contralateral to the deprived eye, non-significant); significant asymmetries between the left and right hemisphere for a given brain region are denoted by a red asterisk. Note the asymmetry between the stimulated and visually deprived hyperpallia during recovery sleep, with the stimulated hyperpallium showing steeper slopes, and the symmetric mean increase in the mesopallium. Reproduced with permission from Lesku et al. (2011b).

One study has provided electrophysiological evidence for local sleep homeostasis in birds (Lesku et al., 2011b). Lesku et al. (2011b) implanted electrodes over the left and right hyperpallia (a primary visual area) and left and right mesopallia (a non-visual processing area). The left eye of each bird was capped, while the right eye was directed toward a monitor showing videos of wild birds (Figure 2A), during which they were prevented from having their normal daytime naps in either hemisphere. In this way, the left hyperpallia, which receives projections primarily from the right eye, was stimulated more than the right hyperpallia, which received reduced visual input, but both hemispheres were kept similarly awake. At the end of the day, the cap was removed and the birds were allowed to sleep undisturbed. During recovery sleep, the left and right mesopallium showed an increase in SWA relative to the baseline night (Figure 2B); the increase was symmetric because the level of SWA was determined only by the amount of wakefulness during the previous day. Conversely, there was an asymmetry in SWA in the hyperpallium with only the stimulated hyperpallium showing an increase. The absence of an increase in SWA in the unstimulated hyperpallium likely reflects the net effect of competing processes that increase (time awake) and decrease (sensory deprivation) (e.g., Huber et al., 2006) SWA relative to baseline. The slope of NREM sleep slow waves – a proposed correlate of synaptic potentiation induced during wakefulness (Vyazovskiy et al., 2011a) – also showed the greatest increase in the stimulated hyperpallium (Figure 2C). Thus, the intensity of NREM sleep is shaped locally within a hemisphere as a function of the duration and intensity of prior wakefulness.

Finally, it conceivable that sleep is homeostatically regulated at an even more local level in the avian brain. During sleep deprivation in mammals, localized neocortical sleep-related oscillations can intrude into global wakefulness, resulting in deficits in waking performance (Vyazovskiy et al., 2011b; Nir et al., 2017). The same phenomenon might occur in the avian hyperpallium, the most dorsal part of the telencephalon from which the EEG is usually recorded. However, the high-density multiunit or cellular recordings of the hyperpallium needed to differentiate between sleep-like potentials resulting from local sleep-related processes and those arising from other sources during active wakefulness (see Boiko and Bureś, 1985; Yang et al., 2008) have not been performed in birds. In addition, birds provide little opportunity to examine the intrusion of sleep-related oscillations into quiet wakefulness, as they usually transition to NREM sleep within seconds of becoming inactive. Nonetheless, if future studies detect localized sleep-related oscillations intruding into wakefulness, it will be interesting to determine whether they adversely impact neurobehavioral performance, as shown in mammals and predicted by modeling (Lima and Rattenborg, 2007). It will also be important to determine the extent to which such brief, localized sleep can compensate for the loss of global sleep during periods of prolonged wakefulness.

### Slow Wave Origin and Propagation

At any point in time during NREM sleep, different parts of the neocortex may be engaged in the hyperpolarized or depolarized phase of the slow oscillation of neuronal membrane potentials. This local aspect of slow oscillations, and resulting EEG slow waves, arises from the fact that slow oscillations originate from multiple neocortical regions and propagate horizontally across the neocortex as a traveling wave (Massimini et al., 2004; Murphy et al., 2009; Nir et al., 2011). Although the neocortex is capable of generating slow oscillations following recovery from thalamotomy, input from the thalamus plays an important role in the generation of slow waves under normal conditions (Crunelli and Hughes, 2010; Timofeev and Chauvette, 2011; Lemieux et al., 2014). Slow waves typically occur first within layer 5 (a thalamorecipient layer) (Constantinople and Bruno, 2013) and propagate vertically within cortical columns (Chauvette et al., 2010; Capone et al., 2019). Although the mechanisms underlying the layer-specific, horizontal propagation of slow waves have been investigated under anesthesia (Sanchez-Vives and McCormick, 2000; Luczak et al., 2007; Sakata and Harris, 2009; Chauvette et al., 2011; Wester and Contreras, 2012; Reyes-Puerta et al., 2015; Capone et al., 2019), little is known about how slow waves propagate horizontally during natural NREM sleep. The functions (if any) of the traveling aspect of slow waves are poorly understood (Muller et al., 2018).

**FIGURE 3:**
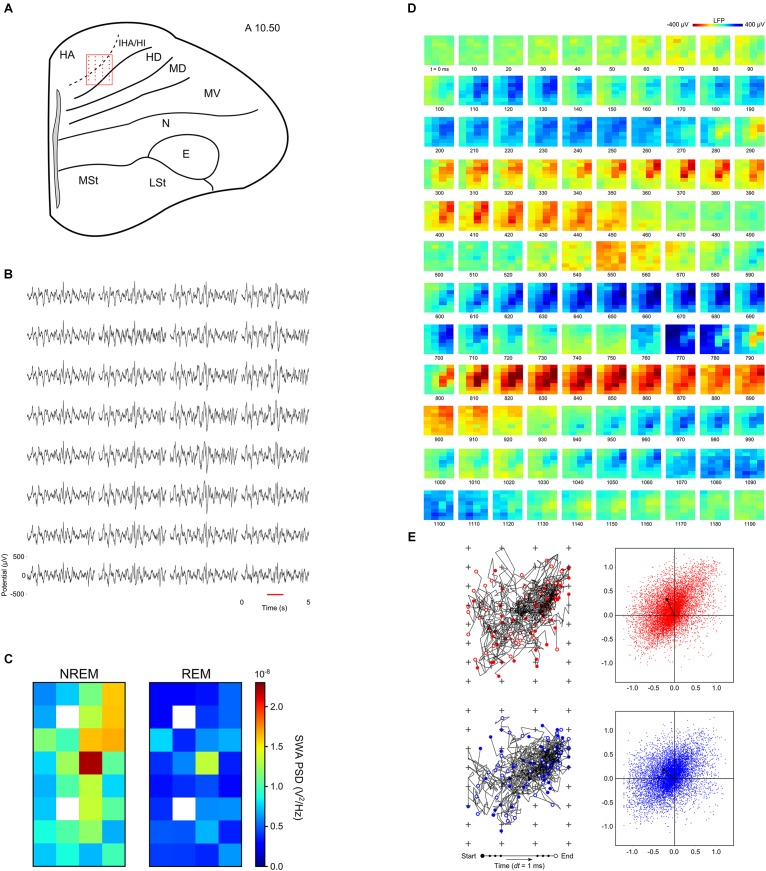
Neurophysiology of the avian hyperpallium during natural sleep. **(A)** Position of a 32-channel silicon electrode probe in the hyperpallium of a pigeon. The orientation of the electrode grid (red) is always depicted with the medial side to the left and the surface of the brain on top. Input from the avian lateral geniculate nucleus (LGN) projects primarily to the interstitial part of hyperpallium apicale (IHA) and the hyperpallium intercalatum (HI). The underlying hyperpallium densocellulare (HD) receives relatively little input from the LGN. The hyperpallium overlies and is interconnected with the dorsal and ventral mesopallium (MD and MV) and nidopallium (N). **(B)** Five-second example of local field potentials showing the spatial distribution of slow waves in the hyperpallium during NREM sleep. **(C)** Mean slow wave activity (SWA; 1.5–4.5 Hz; *N* = 4 birds) over all episodes of NREM and REM sleep reveals greater SWA recorded from the electrodes positioned along the diagonal corresponding to the primary thalamic input layers, IHA and HI. SWA during REM sleep decreases from NREM sleep levels across all layers of the hyperpallium. White squares indicate missing data for some birds. **(D)** Propagating slow waves during NREM sleep. Red underlined episode from panel **(B)** is visualized in a sequence of image plots where pixels represent electrode sites and electrical potential is coded in color. **(E)** Trajectories and net propagation of the negative (red) and positive (blue) components of slow waves from a pigeon during NREM sleep. Left: trajectories for 50 randomly selected negative and positive waves (plus signs depict electrode sites). Right: net wave propagation calculated for every negative and positive wave in a 2 h recording; the black dot shows the mean propagation for negative and positive waves. Panels **(D,E)** demonstrate that both negative and positive potentials propagate most prominently along the thalamic input layers, and slightly into the overlying hyperpallium apicale (HA). E, entopallium; LSt, striatum lateral; MSt, striatum medial. Reproduced with permission from van der Meij et al. (2019a).

Recently, the spatiotemporal properties of slow waves were characterized in the avian hyperpallium (van der Meij et al., 2019a). Most of the hyperpallium is involved in processing visual information and is, in many respects, homologous to the mammalian primary visual cortex (Medina and Reiner, 2000). Like the primary visual cortex, the hyperpallium is composed of layers, some of which receive extensive input from the thalamic lateral geniculate nucleus (Karten et al., 1973; Watanabe et al., 1983). Although the hyperpallium lacks pyramidal neurons with apical dendrites spanning multiple layers, the layers are interconnected via axonal projections (Medina and Reiner, 2000). Van der Meij and colleagues used high-density silicone electrode arrays to record LFP across the various layers of the pigeon’s hyperpallium (Figure 3A). Interestingly, the thalamic input layers – interstitial part of hyperpallium apicale (IHA) and the hyperpallium intercalatum (HI) – play a prominent role in slow waves; slow waves have the highest amplitude within these layers (Figure 3B,C), and they tend to appear first in and propagate through, and outward from, these layers (Figure 3D,E; van der Meij et al., 2019a). Collectively, this suggests that thalamic input might be involved in the genesis of avian slow waves. Alternatively, properties intrinsic to neurons in these layers or the associated cytoarchitecture may favor the initiation and propagation of slow waves. Regardless, this study demonstrates that, as in the mammalian neocortex, slow waves propagate through the avian brain during NREM sleep (see also Beckers et al., 2014).

The mechanisms that mediate avian slow wave propagation remain unclear. Nonetheless, from multi-electrode recordings of anesthesia-induced slow waves, which in many aspects, including propagation pattern, resemble NREM slow waves (van der Meij et al., 2019b), it is clear that the extracellular depolarization of LFP waves is spatio-temporally closely matched by local action potential activity (Beckers et al., 2014), as is the case during NREM sleep in the human neocortex (Nir et al., 2011). Consequently, propagating slow waves appear to reflect the sequential local activation of neurons in the avian brain.

## Local Aspects of REM Sleep

Research on the local aspects of sleep has primarily focused on the cortical manifestation of NREM sleep in mammals and birds. However, in birds, muscle tone during REM sleep also appears to be regulated at a local level. In addition to sleeping with their head turned over the shoulder, birds can also sleep with their head facing forward. Interestingly, when geese sleep with their head facing forward, it slowly drops during REM sleep, presumably reflecting the loss of some tone in the neck muscles (Dewasmes et al., 1985). However, nuchal EMG recordings rarely show a reduction in tone during this behavior; the same behavior and EMG findings have also been reported in several other avian species (e.g., van Twyver and Allison, 1972; Šušić and Kovaèević, 1973; Tobler and Borbély, 1988; Szymczak et al., 1993). Nonetheless, when geese sleep with their head resting fully supported on their back, the same EMG recordings often show hypotonia or atonia (Dewasmes et al., 1985). Importantly, this difference was not due to a lengthening of REM sleep bouts and a resulting progressive reduction in muscle tone when the head was supported, as bouts of REM sleep were similarly short regardless of head position. Consequently, the presence or absence of EMG atonia was specifically related to whether the head was supported or unsupported during REM sleep. Thus, even though the head drops when facing forward, the higher level of neck EMG activity indicates that some tone is maintained, possibly to partially counteract or control the drop. Consistent with the presence of some tone, the drops are usually slow (i.e., not a free-fall) and, in some cases, interrupted by brief halts or jerks (Walker and Berger, 1972; Ayala-Guerrero and Vasconcelos-Dueñas, 1988; Ayala-Guerrero, 1989; Ayala-Guerrero et al., 2003). Collectively, these findings indicate that neck muscle tone is regulated during avian REM sleep to accommodate differing postural demands.

In addition to the neck muscles, birds appear to sustain some tone in the muscles involved in standing during REM sleep. Most mammals lie down to sleep, due, in part, to the loss of skeletal muscle tone during REM sleep. Even horses, giraffes, and elephants, which can stand during NREM sleep, usually engage in REM sleep while lying (Ruckebusch, 1972; Tobler, 1992; Tobler and Schwierin, 1996; see also Gravett et al., 2017). Although horses have a leg locking mechanism (stay apparatus) that reduces the muscular tone needed to stand (Schuurman et al., 2003), apparently this level of tone can only be maintained during NREM sleep. Indeed, when horses are reluctant to lie down, due to pain or perceived vulnerability, they stumble and, in some cases, injure themselves when they enter REM sleep (Aleman et al., 2008; see also Schiffmann et al., 2018). In contrast to horses, birds often engage in REM sleep while standing, even on one foot, without falling (Dewasmes et al., 1985; Szymczak et al., 1993). Several lines of evidence suggest that this is not due to a more effective passive standing mechanism, but rather to the maintenance of some muscle tone during REM sleep. The ability to sleep while standing is often attributed to an “automatic digital flexor mechanism” that causes tendons to pull the foot closed around a perch when the bird relaxes and allows its weight to flex the ankle (Galton and Shepherd, 2012) and a “digital tendon locking mechanism” consisting of interlocking ridges in the digit tendons and overlying tendon sheaths that help to keep the digits closed (Quinn and Baumel, 1990). Some species of bats use a similar digital tendon locking mechanism (Quinn and Baumel, 1993) that allows them to engage in NREM and REM sleep while hanging upside down (Zhao et al., 2010), apparently without any muscular effort, as dead bats are found still hanging (Neuweiler, 2000). However, birds that die in their sleep do not remain perched up-right in trees, and freshly dead or anesthetized starlings are unable to passively grasp a perch (Galton and Shepherd, 2012). Similarly, although some birds may require less muscle tone to balance while standing on the ground on one foot due to the arrangement of the bones in the hip (Stolpe, 1932; Chang and Ting, 2017), it seems likely that some tone is still required to balance (Necker, 2006), let alone to hold one foot up (Necker, 2010). The ability to engage in REM sleep while standing on one foot likely relies on the same mechanisms that enable geese and other birds to maintain neck muscle tone when sleeping with their head unsupported. Finally, similar mechanisms may explain how frigatebirds can engage in brief bouts of REM sleep while soaring (Rattenborg et al., 2016).

The mechanisms mediating the local regulation of muscle tone during avian REM sleep are unknown. In mammals, glutamatergic neurons in the pontine sublaterodorsal nucleus (SLD) project to GABA/glycine inhibitory neurons in the ventromedial medulla (vmM) which in turn hyperpolarize somatic motor neurons resulting in atonia of the postural muscles (Valencia Garcia et al., 2018). The local regulation of muscle tone in birds is difficult to reconcile with this centralized, top-down model of muscle tone regulation in mammals. Assuming that the same central brainstem pathways also mediate reductions in muscle tone in birds, local competitive processes might modulate the impact that this central signal has on motor neurons and muscle tone depending on the postural demands for sustained tone.

The local regulation of muscle tone during avian REM sleep may have implications for understanding the functions of atonia. Traditionally, atonia is thought to prevent dream-related motor cortex output from causing animals to act out their dreams during REM sleep (Jouvet and Delorme, 1965; Morrison, 1983; Mahowald and Schenck, 2005). According to this hypothesis, the phasic twitches that occur during REM sleep are thought to reflect momentary failures of the atonia mechanism that prevents dream-related motor cortex output from reaching the muscles. Neurodegeneration of this brainstem mechanism is thought to give rise to REM sleep behavior disorder, a condition wherein people in REM sleep exhibit elevated muscle tone and complex behaviors that have been attributed to the enactment of dreams (Mahowald and Schenck, 2005). However, several lines of evidence indicate that twitches do not reflect a response to REM sleep-related activity in the motor cortex (Blumberg and Plumeau, 2016). First, dreams also occur during NREM sleep (Siclari et al., 2017), when muscle tone is sustained, but they do not result in dream enactment. Second, twitches persist in cats and week-old rats when the motor cortex is disconnected from the brainstem (Marchiafava and Pompeiano, 1964; Villablanca, 1966; Kreider and Blumberg, 2000). Finally, in intact young rats, activity in the motor cortex follows, rather than precedes twitches (Tiriac et al., 2014). Clearly, twitches do not simply reflect output from the motor cortex during REM sleep, but instead output from the brainstem.

Rather than being a functionless byproduct of dreaming, twitches appear to play an important role in the development and perhaps maintenance of the motor cortex. During wakefulness, deliberate movements are accompanied by a corollary discharge that effectively informs the sensorimotor cortex that a movement has been initiated and the resulting sensory feedback (reafference) should be ignored. By contrast, during REM sleep, reafference resulting from twitches generated by the brainstem reaches the sensorimotor cortex where it induces large responses that are thought to play an active role in the development of sensorimotor neural circuits (Tiriac et al., 2014). Atonia is thought to contribute to this process by increasing the signal-to-noise ratio of sensory signals resulting from twitches. In adults, twitches may also update and recalibrate these sensorimotor circuits (Tiriac and Blumberg, 2016).

Becoming completely atonic and twitchy does not seem to pose a problem for very young birds which do not need to maintain up-right postures. However, it is unclear how adult birds reconcile this seemingly fundamental need for atonia and twitching with the local regulation of muscle tone during REM sleep. Presumably, the muscle groups controlling the digits, legs, etc., involved in standing also need to twitch. Indeed, the fact that reduced muscle tone persists, in at least some muscle groups, during certain postures emphasizes its importance; if it did not serve an important function natural selection should have done away with it altogether. Given the presence of atonia in the neck muscles of geese during REM sleep only when the head is supported (Dewasmes et al., 1985), it is conceivable that the muscles involved in standing become atonic and twitch when birds engage in REM sleep while sitting down. In effect, just as birds can engage in NREM sleep with one hemisphere at a time, they might be able to determine when REM sleep functions attributed to atonia and twitching take place in different parts of the body. Determining how birds reconcile the seemingly competing demands for postural control on the one hand, with atonia and twitching, on the other, might provide novel perspectives on the mechanisms and functions of REM sleep-related atonia and twitching, in general.

## Mixed NREM/REM Sleep States

The heterogeneity of states is not limited to the combination of wakefulness and NREM sleep, in the case of unihemispheric sleep, or in topographic variations in SWA during NREM sleep or REM sleep-related muscle tone. Comparative research reveals that mixed states can arise through the combination of aspects of NREM sleep occurring simultaneously with features of REM sleep. The first evidence for such mixture was gleaned from several studies on monotreme mammals. Owing to the retention of ancestral traits, such as egg-laying (Warren et al., 2008), monotremes might also retain ancestral sleep traits that would have been present in the most recent common ancestor to all mammals. Several studies of sleeping monotremes revealed only cortical slow waves typical of NREM sleep (Allison et al., 1972; Siegel et al., 1996, 1999; but see Nicol et al., 2000). However, concurrent with EEG signs of NREM sleep, were REM sleep-like brainstem unit activity in the short-beaked echidna (*Tachyglossus aculeatus*; Siegel et al., 1996) and REM sleep-related, brainstem-generated phenomena, including REMs and reduced muscle tone in the platypus (*Ornithorhynchus anatinus*; Siegel et al., 1999). The temporal integration and spatial segregation of NREM and REM sleep in the brains of monotremes, suggested that this was the ancestral condition, wherein REM sleep first appeared in the brainstem of early mammals, and later extended to the cortex in the common ancestor to metatherian (marsupial) and eutherian (placental) mammals (Siegel et al., 1998). This hypothesis was strengthened by the absence of similar activity in the brainstem of sleeping turtles (Eiland et al., 2001).

**FIGURE 4:**
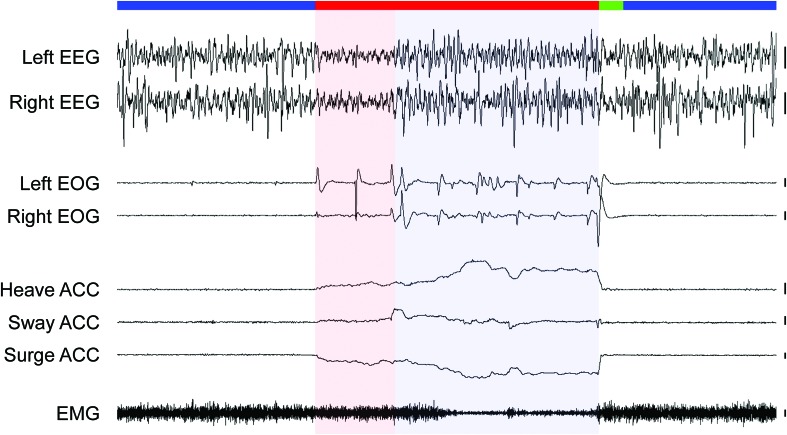
Sleep states in an ostrich. The recording begins and ends with periods of NREM sleep (blue bar) characterized by high amplitude, slow waves in the electroencephalogram (EEG), the absence of rapid eye movements (measured via electrooculogram, EOG), and head movements (accelerometer, ACC), and moderate neck muscle tone (electromyogram, EMG). NREM sleep is interrupted by a period of REM sleep (red bar) with either EEG activation (red shading) or slow waves (blue shading). Irrespective of the type of EEG activity, rapid eye movements, a forward falling and swaying head with moderate-to-low muscle tone occurred invariably during REM sleep in the ostrich. Heave ACC: movement along the dorso-ventral axis with an upward slope denoting downward movement, Sway ACC: lateral axis with up denoting movement to the right, Surge ACC: anterior-posterior axis with down denoting movement forward. Vertical bars to the right of each EEG, EOG, and EMG trace denote 100 μV, and 100 mg-forces to the right of each ACC trace. Trace duration: 60 s. Reproduced with permission from Lesku et al. (2011a).

Motivated by the research on monotremes, Lesku et al. (2011a) investigated sleep in a Palaeognath bird. First, phylogenetically, there are two main groups of living birds: the clade Palaeognathae includes the large flightless ratites (e.g., ostriches, emus, rheas, and cassowaries), small flightless kiwi, and small volant tinamous. The sister clade, Neognathae, includes all other birds. Like monotremes, the Palaeognaths retain ancestral traits, including their reptilian palate anatomy, not found in Neognaths (Mitchell et al., 2014). Interestingly, in addition to a REM sleep state with cortical activation typical of other birds, ostriches (*Struthio camelus*) also have a monotreme-like sleep state characterized by EEG slow waves occurring in conjunction with REMs under closed eyelids, and behavioral and physiological signs of reduced neck muscle tone (Figure 4; Lesku et al., 2011a). This monotreme-like mixed sleep state led Lesku et al. (2011a) to tentatively suggest that the evolution of REM sleep followed a similar trajectory in mammals and birds, occurring first in the brainstem and later becoming exclusively associated with cortical activation. However, unlike other birds, ostriches maintain an unusual sleep posture with the head held periscopically above the ground during unequivocal NREM sleep and entry into this mixed sleep state. Moreover, although the head drops during the mixed state, as a result of reduced muscle tone, the neck remains held off the ground. Consequently, just as geese modulate muscle tone during REM sleep depending on their head position (Dewasmes et al., 1985), it is conceivable that ostriches incorporate features of NREM sleep into REM sleep to maintain some control over their falling head. If true, then such a mixed state might not have any relevance for understanding the evolution of avian sleep states, and we would predict that other Palaeognath birds that sleep with their head better supported (by body or ground) to exhibit distinct sleep states like those observed in Neognath birds (Roth et al., 2006). Moreover, although ostriches exhibit some ancestral traits, they bear little resemblance to early birds, which were small and flighted. For all these reasons, the study of tinamous, which retain the ancestral small size and ability to fly (Mitchell et al., 2014), might be more relevant to understanding how NREM and REM sleep evolved in birds. Unlike their Palaeognath kin, the elegant crested tinamou (*Eudromia elegans*) has sleep states like those of all other birds studied, as characterized by the EEG, EOG, EMG and head movements captured by video recordings and accelerometry (Tisdale et al., 2017). Consequently, it seems that the mixed sleep state described in ostriches does not reflect the ancestral state for birds, but rather a peculiarity of ostriches, perhaps somehow related to their unusual sleep posture.

Indirectly, these findings from two Palaeognath species, also question whether the mixed sleep state found in monotremes reflects the ancestral state for mammals, especially given recent reports of a REM sleep-like state, with wake-like cortical activation, in the bearded dragon lizard (*Pogona vitticeps*) (Shein-Idelson et al., 2016; Libourel et al., 2018). Perhaps even more relevant is the discovery that the cortical correlates of REM sleep-like states vary dramatically, even among different species of lizards recorded using the same methods in the same lab. Unlike bearded dragons, the Argentine tegu (*Salvator merianae*) does not exhibit wake-like cortical activation during putative REM sleep, but rather a novel beta rhythm, rarely observed during wakefulness (Libourel et al., 2018). Collectively, these studies suggest that the cortical correlates of (ostensibly) REM sleep in non-avian reptiles are more diverse than previously recognized, an exciting finding that complicates attempts to draw simple stories for the evolution of sleep states in vertebrates.

Recent studies suggest that slow waves occurring during REM sleep might be more common than previously thought, even in more traditional mammalian models for investigating sleep, such as rodents and humans. Intracortical records from mice revealed that slow waves persist in the thalamic input layers of primary sensory cortices during REM sleep (Funk et al., 2016). By contrast, secondary/association cortices show LFP activation across all layers. Recently, in a high-density EEG study of humans, slow waves were also recorded from at least some primary cortices (Bernardi et al., 2019). Funk et al. (2016) suggest that slow waves occurring in the input layers of primary sensory cortices might gate sensory input, and thereby explain why arousal thresholds are elevated during REM sleep. These studies demonstrate that cortical slow waves can occur during mammalian REM sleep, and thereby question whether this phenomenon explains the presence of slow waves in monotremes exhibiting signs of REM sleep. However, as the EEG electrodes were placed over the parietal and sensorimotor cortex in echidnas (Siegel et al., 1996) and motor cortex in platypuses (Siegel et al., 1999), it is unclear whether slow waves in monotremes and mice reflect the same phenomena. High density EEG and intracortical recordings are needed in monotremes and more placental species, to clarify the generality and potential functions of slow waves during REM sleep.

Recent intra-“cortical” recordings from pigeons demonstrate that slow waves occurring in the thalamic input layers of primary sensory areas is not a universal feature of animals with REM sleep. As described above, during NREM sleep, slow waves in the visual hyperpallium – the avian homolog of the mammalian primary visual cortex – are most evident in thalamic input layers. However, during REM sleep, these layers show a large reduction in slow wave power from prior NREM sleep levels (Figure 3C). Furthermore, in some recordings, these input layers also exhibit bursts of 70–90 Hz gamma activity during REM sleep (van der Meij et al., 2019a). These finding indicate that thalamic input layers of primary sensory cortices do not always exhibit slow waves during REM sleep. In addition, the intra-“cortical” recordings from pigeons indicate that the presence of EEG slow waves occurring in conjunction with signs of REM sleep in ostriches (Lesku et al., 2011a) cannot be attributed to the phenomenon described in mice (Funk et al., 2016). As in most studies of birds, the EEG was recorded from the visual hyperpallium in ostriches, but only ostriches show slow waves occurring in conjunction with other signs of REM sleep. Consequently, slow waves during REM sleep reflect a unique feature of the ostrich hyperpallium, rather than a general feature of the avian hyperpallium during REM sleep.

## Conclusion

In several respects, the local aspects of sleep are similar in mammals and birds. Nonetheless, there are also some potentially informative differences. In both groups, at least some species mitigate the simultaneous ecological need to be awake and the physiological need to sleep by engaging in NREM sleep unihemispherically. Although the evidence for sleep homeostasis at the hemispheric level remains somewhat equivocal, the presence of local use-dependent homeostasis within a hemisphere suggests that homeostasis at the hemispheric level is likely. In both mammals and birds, thalamic input layers play a prominent role in the initiation of propagating slow waves. Despite the similar local aspects of NREM sleep, birds exhibit local aspects of REM sleep not found in mammals. Although birds show visible signs of reduced tone in the muscles supporting the head during REM sleep, the muscles involved in maintaining a standing posture (even on one foot) rarely show reductions in tone during REM sleep. In addition, birds are able to modulate tone in the neck muscles to accommodate different head positions during REM sleep. Like monotreme mammals, ostriches exhibit a mixed sleep state characterized by cortical slow waves occurring with brainstem-mediated features of REM sleep. However, comparisons with other birds and reptiles, challenge the hypothesis that this mixed sleep state reflects an ancestral form of sleep in mammals or birds. Instead, an emerging pattern from recent comparative work is that the EEG correlates of REM sleep may be far more diverse than previously recognized: ostriches have slow waves, but their close tinamou relatives have activation typical of other birds; pogona lizards have wake-like cortical activation, but tegu lizards exhibit a beta rhythm rarely found during wakefulness; and mice exhibit slow waves in the thalamic input layers of the primary visual cortex, but pigeons show activation in these layers. We suspect that as the diversity of species and the number of brain regions examined increases, our ability to package sleep into two, simple, mutually exclusive states will become even more difficult. Although this may tax our ability to define sleep, this diversity also serves as a rich resource for exploring the mechanisms and functions of sleep-related brain activity.

## Author Contributions

NR and JL wrote the initial draft of the manuscript. All authors provided the input on the manuscript. JL and JvdM contributed to the figures.

## Conflict of Interest Statement

The authors declare that the research was conducted in the absence of any commercial or financial relationships that could be construed as a potential conflict of interest.

## References

[B1] Aleman M. Williams D. C. Holliday T. (2008). Sleep and sleep disorders in horses. *AAEP Proc.* 54 180–185.

[B2] Allison T. van Twyver H. Goff W. R. (1972). Electrophysiological studies of the echidna, Tachyglossus aculeatus. I. Waking and sleep. *Arch. Ital. Biol.* 110 145–184. 10.4449/aib.v110i2.2469 4342268

[B3] Astori S. Wimmer R. D. Lüthi A. (2013). Manipulating sleep spindles–expanding views on sleep, memory, and disease. *Trends Neurosci.* 36 738–748. 10.1016/j.tins.2013.10.001 24210901

[B4] Ayala-Guerrero F. (1989). Sleep patterns in the parakeet Melopsittacus undulatus. *Physiol. Behav.* 46 787–791. 10.1016/0031-9384(89)90038-3 2628990

[B5] Ayala-Guerrero F. Mexicano G. Ramos J. I. (2003). Sleep characteristics in the turkey *Meleagris gallopavo*. *Physiol. Behav.* 78 435–440. 10.1016/S0031-9384(03)00032-5 12676279

[B6] Ayala-Guerrero F. Vasconcelos-Dueñas I. (1988). Sleep in the dove *Zenaida asiatica*. *Behav. Neural. Biol.* 49 133–138. 10.1016/S0163-1047(88)90451-7 3365182

[B7] Ball N. J. Amlaner C. J. Shaffery J. P. Opp M. R. (1988). “Asynchronous eye closure and unihemispheric quiet sleep of birds,” in *Sleep 86*, eds Koella W. P. Obál F. Schulz H. Visser P. (New York, NY: Gustav Fischer), 151–153.

[B8] Beckers G. J. L. van der Meij J. Lesku J. A. Rattenborg N. C. (2014). Plumes of neuronal activity propagate in three dimensions through the nuclear avian brain. *BMC Biol.* 12:16. 10.1186/1741-7007-12-16 24580797 PMC4015294

[B9] Bernardi G. Betta M. Ricciardi E. Pietrini P. Tononi G. Siclari F. (2019). Regional delta waves in human rapid-eye movement sleep. *J. Neurosci.* 39 2686–2697. 10.1523/JNEUROSCI.2298-18.2019 30737310 PMC6445986

[B10] Blumberg M. S. Plumeau A. M. (2016). A new view of ”dream enactment” in REM sleep behavior disorder. *Sleep Med. Rev.* 30 34–42. 10.1016/j.smrv.2015.12.002 26802823 PMC4912466

[B11] Boerema A. S. Riedstra B. Strijkstra A. M. (2003). Decrease in monocular sleep after sleep deprivation in the domestic chicken. *Behaviour* 140 1415–1420. 10.1163/156853903771980657

[B12] Boiko V. P. Bureś J. (1985). Electrical phenomena in the telencephalon of the pigeon during pecking. *Neurosci. Behav. Physiol.* 15 265–270. 4033923 10.1007/BF01182997

[B13] Branstetter B. K. Finneran J. J. Fletcher E. A. Weisman B. C. Ridgway S. H. (2012). Dolphins can maintain vigilant behavior through echolocation for 15 days without interruption or cognitive impairment. *PLoS One* 7:e47478. 10.1371/journal.pone.0047478 23082170 PMC3474785

[B14] Brusatte S. L. O’Connor J. K. Jarvis E. D. (2015). The origin and diversification of birds. *Curr. Biol.* 25 R888–R898. 10.1016/j.cub.2015.08.003 26439352

[B15] Capone C. Rebollo B. Muñoz A. Illa X. Del Giudice P. Sanchez-Vives M. V. (2019). Slow waves in cortical slices: how spontaneous activity is shaped by laminar structure. *Cereb. Cortex* 9 319–335. 10.1093/cercor/bhx326 29190336

[B16] Castelnovo A. Lopez R. Proserpio P. Nobili L. Dauvilliers Y. (2018). NREM sleep parasomnias as disorders of sleep-state dissociation. *Nat. Rev. Neurol.* 14 470–481. 10.1038/s41582-018-0030-y 29959394

[B17] Chang Y. H. Ting L. H. (2017). Mechanical evidence that flamingos can support their body on one leg with little active muscular force. *Biol. Lett.* 13 20160948. 10.1098/rsbl.2016.0948 28539457 PMC5454233

[B18] Chauvette S. Crochet S. Volgushev M. Timofeev I. (2011). Properties of slow oscillation during slow-wave sleep and anesthesia in cats. *J. Neurosci.* 31 14998–15008. 10.1523/JNEUROSCI.2339-11.2011 22016533 PMC3209581

[B19] Chauvette S. Volgushev M. Timofeev I. (2010). Origin of active states in local neocortical networks during slow sleep oscillation. *Cereb. Cortex* 20 2660–2674. 10.1093/cercor/bhq009 20200108 PMC2951844

[B20] Constantinople C. M. Bruno R. M. (2013). Deep cortical layers are activated directly by thalamus. *Science* 340 1591–1594. 10.1126/science.1236425 23812718 PMC4203320

[B21] Crunelli V. Hughes S. W. (2010). The slow (< 1 Hz) rhythm of non-REM sleep: a dialogue between three cardinal oscillators. *Nat. Neurosci.* 13 10–18. 10.1038/nn.2445 19966841 PMC2980822

[B22] Dewasmes G. Cohen-Adad F. Koubi H. Le Maho Y. (1985). Polygraphic and behavioral study of sleep in geese: existence of nuchal atonia during paradoxical sleep. *Physiol. Behav.* 35 67–73. 10.1016/0031-9384(85)90173-8 4059402

[B23] Durán E. Oyanedel C. N. Niethard N. Inostroza M. Born J. (2018). Sleep stage dynamics in neocortex and hippocampus. *Sleep* 41:zsy060. 10.1093/sleep/zsy060 29893972

[B24] Eiland M. M. Lyamin O. I. Siegel J. M. (2001). State-related discharge of neurons in the brainstem of freely moving box turtles, *Terrapene carolina* major. *Arch. Ital. Biol.* 139 23–36. 10.4449/aib.v139i1.202 11256184 PMC9048152

[B25] Emrick J. J. Gross B. A. Riley B. T. Poe G. R. (2016). Different simultaneous sleep states in the hippocampus and neocortex. *Sleep* 39 2201–2209. 10.5665/sleep.6326 27748240 PMC5103808

[B26] Fuchs T. Maury D. Moore F. R. Bingman V. P. (2009). Daytime micro-naps in a nocturnal migrant: an EEG analysis. *Biol. Lett.* 5 77–80. 10.1098/rsbl.2008.0405 18990656 PMC2657733

[B27] Funk C. M. Honjoh S. Rodriguez A. V. Cirelli C. Tononi G. (2016). Local slow waves in superficial layers of primary cortical areas during REM sleep. *Curr. Biol.* 26 396–403. 10.1016/j.cub.2015.11.062 26804554 PMC4747819

[B28] Galton P. M. Shepherd J. D. (2012). Experimental analysis of perching in the European starling (*Sturnus vulgaris*: Passeriformes; Passeres), and the automatic perching mechanism of birds. *J. Exp. Zool. A Ecol. Genet. Physiol.* 317 205–215. 10.1002/jez.1714 22539208

[B29] Gill R. E. Tibbitts T. L. Douglas D. C. Handel C. M. Mulcahy D. M. Gottschalck J. C. (2009). Extreme endurance flights by landbirds crossing the Pacific Ocean: ecological corridor rather than barrier? *Proc. R. Soc. B* 276 447–458. 10.1098/rspb.2008.1142 18974033 PMC2664343

[B30] Gnone G. Moriconi T. Gambini G. (2006). Sleep behaviour: activity and sleep in dolphins. *Nature* 441 E10–E11. 10.1038/nature04899 16791148

[B31] Goley P. D. (1999). Behavioral aspects of sleep in Pacific white-sided dolphins (*Lagenorhynchus obliquidens*, Gill 1865). *Mar. Mam. Sci.* 15 1054–1064. 10.1111/j.1748-7692.1999.tb00877.x

[B32] Graf R. Heller H. C. Sakaguchi S. Krishna S. (1987). Influence of spinal and hypothalamic warming on metabolism and sleep in pigeons. *Am. J. Physiol.* 252 R661–R667. 10.1152/ajpregu.1987.252.4.R661 3565598

[B33] Gravett N. Bhagwandin A. Sutcliffe R. Landen K. Chase M. J. Lyamin O. I. (2017). Inactivity/sleep in two wild free-roaming African elephant matriarchs - Does large body size make elephants the shortest mammalian sleepers? *PLoS One* 12:e0171903. 10.1371/journal.pone.0171903 28249035 PMC5382951

[B34] Hanlon E. C. Faraguna U. Vyazovskiy V. V. Tononi G. Cirelli C. (2009). Effects of skilled training on sleep slow wave activity and cortical gene expression in the rat. *Sleep* 32 719–729. 10.1093/sleep/32.6.719 19544747 PMC2690558

[B35] Hedenström A. Norevik G. Warfvinge K. Andersson A. Bäckman J. Åkesson S. (2016). Annual 10-month aerial life phase in the common swift *Apus apus*. *Curr. Biol.* 26 1–5. 10.1016/j.cub.2016.09.014 28094028

[B36] Heller H. C. Graf R. Rautenberg W. (1983). Circadian and arousal state influences on thermoregulation in the pigeon. *Am. J. Physiol.* 245 R321–R328. 10.1152/ajpregu.1983.245.3.R321 6614203

[B37] Huber R. Ghilardi M. F. Massimini M. Ferrarelli F. Riedner B. A. Peterson M. J. (2006). Arm immobilization causes cortical plastic changes and locally decreases sleep slow wave activity. *Nat. Neurosci.* 9 1169–1176. 10.1038/nn1758 16936722

[B38] Huber R. Ghilardi M. F. Massimini M. Tononi G. (2004). Local sleep and learning. *Nature* 430 78–81. 10.1038/nature02663 15184907

[B39] Jones S. G. Vyazovskiy V. V. Cirelli C. Tononi G. Benca R. M. (2008). Homeostatic regulation of sleep in the white-crowned sparrow (*Zonotrichia leucophrys gambelii*). *BMC Neurosci.* 9:47. 10.1186/1471-2202-9-47 18505569 PMC2424059

[B40] Jouvet M. Delorme F. (1965). Locus Coeruleus et Sommeil Paradoxal. *C. R. Seances Soc. Biol. Fil.* 159 895–899. 4221672

[B41] Jouvet-Mounier D. Astic L. Lacote D. (1970). Ontogenesis of the states of sleep in rat, cat, and guinea pig during the first postnatal month. *Dev. Psychobiol.* 2 216–239. 10.1002/dev.420020407 5527153

[B42] Karten H. J. Hodos W. Nauta W. J. Revzin A. M. (1973). Neural connections of the “visual wulst” of the avian telencephalon. Experimental studies in the pigeon (*Columba livia*) and owl (*Speotyto cunicularia*). *J. Comp. Neurol.* 150 253–277. 10.1002/cne.901500303 4721779

[B43] Kattler H. Dijk D. J. Borbély A. A. (1994). Effect of unilateral somatosensory stimulation prior to sleep on the sleep EEG in humans. *J. Sleep Res.* 3 159–164. 10.1111/j.1365-2869.1994.tb00123.x 10607121

[B44] Kelly M. L. Peters R. A. Tisdale R. K. Lesku J. A. (2015). Unihemispheric sleep in crocodilians? *J. Exp. Biol.* 218 3175–3178. 10.1242/jeb.127605 26491191

[B45] Kreider J. C. Blumberg M. S. (2000). Mesopontine contribution to the expression of active ’twitch’ sleep in decerebrate week-old rats. *Brain Res.* 872 149–159. 10.1016/S0006-8993(00)02518-X 10924687

[B46] Krueger J. M. Nguyen J. T. Dykstra-Aiello C. J. Taishi P. (2019). Local sleep. *Sleep Med. Rev.* 43 14–21. 10.1016/j.smrv.2018.10.001 30502497 PMC6351167

[B47] Krueger J. M. Obál F. (1993). A neuronal group theory of sleep function. *J. Sleep Res.* 2 63–69. 10.1111/j.1365-2869.1993.tb00064.x 10607073

[B48] Landsness E. C. Crupi D. Hulse B. K. Peterson M. J. Huber R. Ansari H. (2009). Sleep-dependent improvement in visuomotor learning: a causal role for slow waves. *Sleep* 32 1273–1284. 10.1093/sleep/32.10.1273 19848357 PMC2753806

[B49] Lemieux M. Chen J. Y. Lonjers P. Bazhenov M. Timofeev I. (2014). The impact of cortical deafferentation on the neocortical slow oscillation. *J. Neurosci.* 34 5689–5703. 10.1523/JNEUROSCI.1156-13.2014 24741059 PMC3988418

[B50] Lesku J. A. Meyer L. C. R. Fuller A. Maloney S. K. Dell’Omo G. Vyssotski A. L. (2011a). Ostriches sleep like platypuses. *PLoS One* 6:e23203. 10.1371/journal.pone.0023203 21887239 PMC3160860

[B51] Lesku J. A. Vyssotski A. L. Martinez-Gonzalez D. Wilzeck C. Rattenborg N. C. (2011b). Local sleep homeostasis in the avian brain: convergence of sleep function in mammals and birds? *Proc. R. Soc. B* 278 2419–2428. 10.1098/rspb.2010.2316 21208955 PMC3125620

[B52] Lesku J. A. Rattenborg N. C. Valcu M. Vyssotski A. L. Kuhn S. Kuemmeth F. (2012). Adaptive sleep loss in polygynous pectoral sandpipers. *Science* 337 1654–1658. 10.1126/science.1220939 22878501

[B53] Lesku J. A. Roth T. C. Rattenborg N. C. Amlaner C. J. Lima S. L. (2009). History and future of comparative analyses in sleep research. *Neurosci. Biobehav. Rev.* 33 1024–1036. 10.1016/j.neubiorev.2009.04.002 19443034

[B54] Libourel P. A. Barrillot B. Arthaud S. Massot B. Morel A. L. Beuf O. (2018). Partial homologies between sleep states in lizards, mammals, and birds suggest a complex evolution of sleep states in amniotes. *PLoS Biol.* 16:e2005982. 10.1371/journal.pbio.2005982 30307933 PMC6181266

[B55] Liechti F. Witvliet W. Weber R. Bächler E. (2013). First evidence of a 200-day non-stop flight in a bird. *Nat. Commun.* 4:2554. 10.1038/ncomms3554 24104955

[B56] Lima S. L. Rattenborg N. C. (2007). A behavioural shutdown can make sleeping safer: a strategic perspective on the function of sleep. *Anim. Behav.* 74 189–197. 10.1016/j.anbehav.2006.12.007

[B57] Low P. S. Shank S. S. Sejnowski T. J. Margoliash D. (2008). Mammalian-like features of sleep structure in zebra finches. *Proc. Natl. Acad. Sci. U.S.A.* 105 9081–9086. 10.1073/pnas.0703452105 18579776 PMC2440357

[B58] Luczak A. Barthó P. Marguet S. L. Buzsáki G. Harris K. D. (2007). Sequential structure of neocortical spontaneous activity in vivo. *Proc. Natl. Acad. Sci. U.S.A.* 104 347–352. 10.1073/pnas.0605643104 17185420 PMC1765463

[B59] Lyamin O. Pryaslova J. Kosenko P. Siegel J. (2007). Behavioral aspects of sleep in bottlenose dolphin mothers and their calves. *Physiol. Behav.* 92 725–733. 10.1016/j.physbeh.2007.05.064 17599365 PMC8142817

[B60] Lyamin O. I. Kosenko P. O. Korneva S. M. Vyssotski A. L. Mukhametov L. M. Siegel J. M. (2018). Fur seals suppress REM sleep for very long periods without subsequent rebound. *Curr. Biol.* 28 2000–2005. 10.1016/j.cub.2018.05.022 29887309 PMC8670325

[B61] Lyamin O. I. Kosenko P. O. Lapierre J. L. Mukhametov L. M. Siegel J. M. (2008c). Fur seals display a strong drive for bilateral slow-wave sleep while on land. *J. Neurosci.* 28 12614–12621. 10.1523/JNEUROSCI.2306-08.2008 19036955 PMC6671816

[B62] Lyamin O. I. Kosenko P. O. Vyssotski A. L. Lapierre J. L. Siegel J. M. Mukhametov L. M. (2012). Study of sleep in a walrus. *Dokl. Biol. Sci.* 444 188–191. 10.1134/S0012496612030143 22760621 PMC8741258

[B63] Lyamin O. I. Lapierre J. L. Kosenko P. O. Kodama T. Bhagwandin A. Korneva S. M. (2016). Monoamine release during unihemispheric sleep and unihemispheric waking in the fur seal. *Sleep* 39 625–636. 10.5665/sleep.5540 26715233 PMC4763370

[B64] Lyamin O. I. Lapierre J. L. Kosenko P. O. Mukhametov L. M. Siegel J. M. (2008a). Electroencephalogram asymmetry and spectral power during sleep in the northern fur seal. *J. Sleep Res.* 17 154–165. 10.1111/j.1365-2869.2008.00639.x 18482104

[B65] Lyamin O. I. Manger P. R. Ridgway S. H. Mukhametov L. M. Siegel J. M. (2008b). Cetacean sleep: an unusual form of mammalian sleep. *Neurosci. Biobehav. Rev.* 32 1451–1484. 10.1016/j.neubiorev.2008.05.023 18602158 PMC8742503

[B66] Lyamin O. I. Mukhametov L. M. Siegel J. M. (2004). Relationship between sleep and eye state in Cetaceans and Pinnipeds. *Arch. Ital. Biol.* 142 557–568. 10.4449/aib.v142i4.427 15493557 PMC8767446

[B67] Lyamin O. I. Mukhametov L. M. Siegel J. M. Nazarenko E. A. Polyakova I. G. Shpak O. V. (2002). Unihemispheric slow wave sleep and the state of the eyes in a white whale. *Behav. Brain Res.* 129 125–129. 10.1016/S0166-4328(01)00346-1 11809503 PMC8788623

[B68] Mahowald M. Schenck C. (2005). Insights from studying human sleep disorders. *Nature* 437 1279–1285. 10.1038/nature04287 16251953

[B69] Marchiafava P. L. Pompeiano O. (1964). Pyramidal influences of spinal cord during desynchronized sleep. *Arch. Ital. Biol.* 102 500–529. 10.1007/BF00364696 14196087

[B70] Martinez-Gonzalez D. Lesku J. A. Rattenborg N. C. (2008). Increased EEG spectral power density during sleep following short-term sleep deprivation in pigeons (*Columba livia*): evidence for avian sleep homeostasis. *J. Sleep Res.* 17 140–153. 10.1111/j.1365-2869.2008.00636.x 18321247

[B71] Mascetti G. G. (2016). Unihemispheric sleep and asymmetrical sleep: behavioral, neurophysiological, and functional perspectives. *Nat. Sci. Sleep* 8 221–238. 10.2147/NSS.S71970 27471418 PMC4948738

[B72] Mascetti G. G. Rugger M. Vallortigara G. Bobbo D. (2007). Monocular-unihemispheric sleep and visual discrimination learning in the domestic chick. *Exp. Brain Res.* 176 70–84. 10.1007/s00221-006-0595-3 16874518

[B73] Mascetti G. G. Vallortigara G. (2001). Why do birds sleep with one eye open? Light exposure of the chick embryo as a determinant of monocular sleep. *Curr. Biol.* 11 971–974. 10.1016/S0960-9822(01)00265-2 11448774

[B74] Massimini M. Huber R. Ferrarelli F. Hill S. Tononi G. (2004). The sleep slow oscillation as a traveling wave. *J. Neurosci.* 24 6862–6870. 10.1523/JNEUROSCI.1318-04.2004 15295020 PMC6729597

[B75] Mathews C. G. Lesku J. A. Lima S. L. Amlaner C. J. (2006). Asynchronous eye closure as an anti-predator behavior in the western fence lizard (*Sceloporus occidentalis*). *Ethology* 112 286–292. 10.1111/j.1439-0310.2006.01138.x

[B76] Medina L. Reiner A. (2000). Do birds possess homologues of mammalian primary visual, somatosensory and motor cortices? *Trends Neurosci*. 23 1–12. 10.1016/S0166-2236(99)01486-1 10631781

[B77] Mitchell K. J. Llamas B. Soubrier J. Rawlence N. J. Worthy T. H. Wood J. (2014). Ancient DNA reveals elephant birds and kiwi are sister taxa and clarifies ratite bird evolution. *Science* 344 898–900. 10.1126/science.1251981 24855267

[B78] Morrison A. (1983). A window on the sleeping brain. *Sci. Am.* 248 94–102. 10.1038/scientificamerican0483-94 6844908

[B79] Mukhametov L. M. Lyamin O. I. Chetyrbok I. S. Vassilyev A. A. Diaz R. (1992). Sleep in an Amazonian manatee, *Trichechus inunguis*. *Experientia* 48 417–419. 1582500 10.1007/BF01923447

[B80] Mukhametov L. M. Supin A. Y. Polyakova I. G. (1977). Interhemispheric asymmetry of the electroencephalographic sleep patterns in dolphins. *Brain Res.* 134 581–584. 10.1016/0006-8993(77)90835-6 902119

[B81] Muller L. Chavane F. Reynolds J. Sejnowski T. J. (2018). Cortical travelling waves: mechanisms and computational principles. *Nat. Rev. Neurosci.* 19 255–268. 10.1038/nrn.2018.20 29563572 PMC5933075

[B82] Murphy M. Riedner B. A. Huber R. Massimini M. Ferrarelli F. Tononi G. (2009). Source modeling sleep slow waves. *Proc. Natl. Acad. Sci. U.S.A.* 106 1608–1613. 10.1073/pnas.0807933106 19164756 PMC2635823

[B83] Necker R. (2006). Specializations in the lumbosacral vertebral canal and spinal cord of birds: evidence of a function as a sense organ which is involved in the control of walking. *J. Comp. Physiol. A. Neuroethol. Sens. Neural Behav. Physiol.* 192 439–448. 10.1007/s00359-006-0105-x 16450117

[B84] Necker R. (2010). Stehen der Vögel auf einem Bein: mechanismen und mögliche Funktionen - eine Übersicht (Birds standing on one leg: mechanisms and possible functions - a review). *Vogelwarte* 48 43–44.

[B85] Nelini C. Bobbo D. Mascetti G. G. (2010). Local sleep: a spatial learning task enhances sleep in the right hemisphere of domestic chicks (*Gallus gallus*). *Exp. Brain Res.* 205 195–204. 10.1007/s00221-010-2352-x 20625703

[B86] Nelini C. Bobbo D. Mascetti G. G. (2012). Monocular learning of a spatial task enhances sleep in the right hemisphere of domestic chicks (*Gallus gallus*). *Exp. Brain Res.* 218 381–388. 10.1007/s00221-012-3023-x 22349558

[B87] Neuweiler G. (2000). *The Biology of Bats.* Oxford: Oxford University Press.

[B88] Newman S. M. Paletz E. M. Rattenborg N. C. Obermeyer W. H. Benca R. M. (2008). Sleep deprivation in the pigeon using the disk-over-water method. *Physiol. Behav.* 93 50–58. 10.1016/j.physbeh.2007.07.012 17765274

[B89] Nicol S. C. Andersen N. A. Phillips N. H. Berger R. J. (2000). The echidna manifests typical characteristics of rapid eye movement sleep. *Neurosci. Lett.* 283 49–52. 10.1016/S0304-3940(00)00922-8 10729631

[B90] Nir Y. Andrillon T. Marmelshtein A. Suthana N. Cirelli C. Tononi G. (2017). Selective neuronal lapses precede human cognitive lapses following sleep deprivation. *Nat. Med.* 23 1474–1480. 10.1038/nm.4433 29106402 PMC5720899

[B91] Nir Y. Staba R. J. Andrillon T. Vyazovskiy V. V. Cirelli C. Fried I. (2011). Regional slow waves and spindles in human sleep. *Neuron* 70 153–169. 10.1016/j.neuron.2011.02.043 21482364 PMC3108825

[B92] Oleksenko A. I. Mukhametov L. M. Polyakova I. G. Supin A. Y. Kovalzon V. M. (1992). Unihemispheric sleep deprivation in bottlenose dolphins. *J. Sleep Res.* 1 40–44. 10.1111/j.1365-2869.1992.tb00007.x 10607024

[B93] Ookawa T. (2004). The electroencephalogram and sleep in the domestic chicken. *Avian Poult. Biol. Rev.* 15 1–8. 10.3184/147020604783637453

[B94] Ookawa T. Takagi K. (1968). Electroencephalograms of free behavioral chicks at various developmental ages. *Jpn. J. Physiol.* 18 87–99. 5302356 10.2170/jjphysiol.18.87

[B95] Peters J. Vonderahe A. Schmid D. (1965). Onset of cerebral electrical activity associated with behavioral sleep and attention in the developing chick. *J. Exp. Zool.* 160 255–262. 5884103 10.1002/jez.1401600303

[B96] Quercia A. Bobbo D. Mascetti G. G. (2018). The effect of monocular deprivation on unihemispheric sleep in light and dark incubated/reared domestic chicks. *Laterality* 23 166–183. 10.1080/1357650X.2017.1347180 28670970

[B97] Quinn T. H. Baumel J. J. (1990). The digital tendon locking mechanism of the avian foot (Aves). *Zoomorphology* 109 281–293. 10.1007/BF00312195 22539208

[B98] Quinn T. H. Baumel J. J. (1993). Chiropteran tendon locking mechanism. *J. Morph.* 216 197–208. 10.1002/jmor.1052160207 8515478

[B99] Rattenborg N. C. (2017). Sleeping on the wing. *Interface Focus* 7:20160082. 10.1098/rsfs.2016.0082 28163874 PMC5206601

[B100] Rattenborg N. C. Amlaner C. J. Lima S. L. (2000). Behavioral, neurophysiological and evolutionary perspectives on unihemispheric sleep. *Neurosci. Biobehav. Rev.* 24 817–842. 10.1016/S0149-7634(00)00039-7 11118608

[B101] Rattenborg N. C. Amlaner C. J. Lima S. L. (2001). Unilateral eye closure and interhemispheric EEG asymmetry during sleep in the pigeon (*Columba livia*). *Brain Behav. Evol.* 58 323–332. 10.1159/000057573 12016351

[B102] Rattenborg N. C. Lima S. L. Amlaner C. J. (1999a). Facultative control of avian unihemispheric sleep under the risk of predation. *Behav. Brain Res.* 105 163–172. 10.1016/S0166-4328(99)00070-4 10563490

[B103] Rattenborg N. C. Lima S. L. Amlaner C. J. (1999b). Half-awake to the risk of predation. *Nature* 397 397–398. 10.1038/17037 29667967

[B104] Rattenborg N. C. Lima S. L. Lesku J. A. (2012). Sleep locally, act globally. *Neuroscientist* 18 533–546. 10.1177/1073858412441086 22572533

[B105] Rattenborg N. C. Mandt B. H. Obermeyer W. H. Winsauer P. J. Huber R. Wikelski M. (2004). Migratory sleeplessness in the white-crowned sparrow (*Zonotrichia leucophrys gambelii*). *PloS Biol.* 2:E212. 10.1371/journal.pbio.0020212 15252455 PMC449897

[B106] Rattenborg N. C. Martinez-Gonzalez D. Lesku J. A. (2009). Avian sleep homeostasis: convergent evolution of complex brains, cognition and sleep functions in mammals and birds. *Neurosci. Biobehav. Rev.* 33 253–270. 10.1016/j.neubiorev.2008.08.010 18789355

[B107] Rattenborg N. C. Martinez-Gonzalez D. Roth T. C. Pravosudov V. V. (2011). Hippocampal memory consolidation during sleep: a comparison of mammals and birds. *Biol. Rev. Camb. Philos. Soc.* 86 658–691. 10.1111/j.1469-185X.2010.00165.x 21070585 PMC3117012

[B108] Rattenborg N. C. Voirin B. Cruz S. M. Tisdale R. Dell’Omo G. Lipp H.-P. (2016). Evidence that birds sleep in mid-flight. *Nat. Commun.* 7:12468. 10.1038/ncomms12468 27485308 PMC4976198

[B109] Reiner A. Stern E. A. Wilson C. J. (2001). Physiology and morphology of intratelencephalically projecting corticostriatal-type neurons in pigeons as revealed by intracellular recording and cell filling. *Brain Behav. Evol.* 58 101–114. 10.1159/000047264 11805376

[B110] Reyes-Puerta V. Sun J. J. Kim S. Kilb W. Luhmann H. J. (2015). Laminar and columnar structure of sensory-evoked multineuronal spike sequences in adult rat barrel cortex in vivo. *Cereb. Cortex* 25 2001–2021. 10.1093/cercor/bhu007 24518757

[B111] Ridgway S. Carder D. Finneran J. Keogh M. Kamolnick T. Todd M. (2006). Dolphin continuous auditory vigilance for five days. *J. Exp. Biol.* 209 3621–3628. 10.1242/jeb.02405 16943502

[B112] Ridgway S. Keogh M. Carder D. Finneran J. Kamolnick T. Todd M. (2009). Dolphins maintain cognitive performance during 72 to 120 hours of continuous auditory vigilance. *J. Exp. Biol.* 212 1519–1527. 10.1242/jeb.027896 19411545

[B113] Roffwarg H. P. Muzio J. N. Dement W. C. (1966). Ontogenetic development of the human sleep-dream cycle. *Science* 152 604–619. 10.1126/science.152.3722.604 17779492

[B114] Roth T. C. Lesku J. A. Amlaner C. J. Lima S. L. (2006). A phylogenetic analysis of the correlates of sleep in birds. *J. Sleep Res.* 15 395–402. 10.1111/j.1365-2869.2006.00559.x 17118096

[B115] Ruckebusch Y. (1972). Relevance of drowsiness in circadian cycle of farm animals. *Anim. Behav.* 20 637–643. 10.1016/S0003-3472(72)80136-2 4661312

[B116] Sakata S. Harris K. D. (2009). Laminar structure of spontaneous and sensory-evoked population activity in auditory cortex. *Neuron* 64 404–418. 10.1016/j.neuron.2009.09.020 19914188 PMC2778614

[B117] Sanchez-Vives M. V. McCormick D. A. (2000). Cellular and network mechanisms of rhythmic recurrent activity in neocortex. *Nat. Neurosci.* 3 1027–1034. 10.1038/79848 11017176

[B118] Schiffmann C. Hoby S. Wenker C. Hård T. Scholz R. Clauss M. (2018). When elephants fall asleep: a literature review on elephant rest with case studies on elephant falling bouts, and practical solutions for zoo elephants. *Zoo Biol.* 37 133–145. 10.1002/zoo.21406 29600558

[B119] Schuurman S. O. Kersten W. Weijs W. A. (2003). The equine hind limb is actively stabilized during standing. *J. Anat.* 202 355–362. 10.1046/j.1469-7580.2003.00166.x 12739613 PMC1571089

[B120] Scriba M. F. Ducrest A.-L. Henry I. Vyssotski A. L. Rattenborg N. C. Roulin A. (2013). Linking melanism to brain development: expression of a melanism-related gene in barn owl feather follicles covaries with sleep ontogeny. *Front. Zool.* 10:42. 10.1186/1742-9994-10-42 23886007 PMC3734112

[B121] Sekiguchi Y. Arai K. Kohshima S. (2006). Sleep behaviour: sleep in continuously active dolphins. *Nature* 441 E9–E10. 10.1038/nature04898 16791150

[B122] Shein-Idelson M. Ondracek J. M. Liaw H. P. Reiter S. Laurent G. (2016). Slow waves, sharp waves, ripples, and REM in sleeping dragons. *Science* 352 590–595. 10.1126/science.aaf3621 27126045

[B123] Siclari F. Baird B. Perogamvros L. Bernardi G. LaRocque J. J. Riedner B. (2017). The neural correlates of dreaming. *Nat. Neurosci.* 20 872–878. 10.1038/nn.4545 28394322 PMC5462120

[B124] Siclari F. Tononi G. (2017). Local aspects of sleep and wakefulness. *Curr. Opin. Neurobiol.* 44 222–227. 10.1016/j.conb.2017.05.008 28575720 PMC6445546

[B125] Siegel J. M. (2008). Do all animals sleep? *Trends Neurosci*. 31 208–213. 10.1016/j.tins.2008.02.001 18328577 PMC8765194

[B126] Siegel J. M. Manger P. R. Nienhuis R. Fahringer H. M. Pettigrew J. D. (1996). The echidna *Tachyglossus aculeatus* combines REM and non-REM aspects in a single sleep state: implications for the evolution of sleep. *J. Neurosci.* 16 3500–3506. 10.1523/JNEUROSCI.16-10-03500.1996 8627382 PMC6579141

[B127] Siegel J. M. Manger P. R. Nienhuis R. Fahringer H. M. Pettigrew J. D. (1998). Monotremes and the evolution of rapid eye movement sleep. *Philos. Trans. R. Soc. B* 353 1147–1157. 10.1098/rstb.1998.0272 9720111 PMC1692309

[B128] Siegel J. M. Manger P. R. Nienhuis R. Fahringer H. M. Shalita T. Pettigrew J. D. (1999). Sleep in the platypus. *Neurosci.* 91 391–400. 10.1016/S0306-4522(98)00588-0 PMC8760620 10336087

[B129] Spooner C. E. (1964). *Observations on the Use of the Chick in the Pharmacological Investigation of the Central Nervous System.* Ph. D. dissertation. Los Angeles, CA: University of California.

[B130] Steriade M. (2006). Grouping of brain rhythms in corticothalamic systems. *Neuroscience* 137 1087–1106. 10.1016/j.neuroscience.2005.10.029 16343791

[B131] Steriade M. Nuñez A. Amzica F. (1993). A novel slow (< 1 Hz) oscillation of neocortical neurons in vivo: depolarizing and hyperpolarizing components. *J. Neurosci.* 13 3252–3265. 10.1523/JNEUROSCI.13-08-03252.1993 8340806 PMC6576541

[B132] Stolpe M. (1932). Physiologisch-anatomische untersuchungen über die hintere extremität der vögel. *J. Ornithol.* 80 161–247.

[B133] Šušić V. T. Kovaèević R. M. (1973). Sleep patterns in the owl *Strix aluco*. *Physiol. Behav.* 11 313–317. 10.1016/0031-9384(73)90005-X 4355440

[B134] Szymczak J. T. Helb H. W. Kaiser W. (1993). Electrophysiological and behavioral correlates of sleep in the blackbird (*Turdus merula*). *Physiol. Behav.* 53 1201–1210. 10.1016/0031-9384(93)90380-X 8346306

[B135] Szymczak J. T. Kaiser W. Helb H. W. Beszczyńska B. (1996). A study of sleep in the European blackbird. *Physiol. Behav.* 60 1115–1120. 10.1016/0031-9384(96)00231-4 8884941

[B136] Tamaki M. Bang J. W. Watanabe T. Sasaki Y. (2016). Night watch in one brain hemisphere during sleep associated with the first-night effect in humans. *Curr. Biol.* 26 1190–1194. 10.1016/j.cub.2016.02.063 27112296 PMC4864126

[B137] Tew Kai E. Rossi V. Sudre J. Weimerskirch H. Lopez C. Hernandez-Garcia E. (2009). Top marine predators track Lagrangian coherent structures. *Proc. Natl. Acad. Sci. U.S.A.* 106 8245–8250. 10.1073/pnas.0811034106 19416811 PMC2677090

[B138] Timofeev I. Chauvette S. (2011). Thalamocortical oscillations: local control of EEG slow waves. *Curr. Top. Med. Chem.* 11 2457–2471. 10.2174/156802611797470376 21906018

[B139] Tiriac A. Blumberg M. S. (2016). The case of the disappearing spindle burst. *Neural. Plast.* 2016:8037321. 10.1155/2016/8037321 27119028 PMC4826930

[B140] Tiriac A. Del Rio-Bermudez C. Blumberg M. S. (2014). Self-generated movements with ”unexpected” sensory consequences. *Curr. Biol.* 24 2136–2141. 10.1016/j.cub.2014.07.053 25131675 PMC4175005

[B141] Tisdale R. K. Vyssotski A. L. Lesku J. A. Rattenborg N. C. (2017). Sleep-related electrophysiology and behavior of tinamous (*Eudromia elegans*): tinamous don’t sleep like ostriches. *Brain Behav. Evol.* 89 249–261. 10.1159/000475590 28683451

[B142] Tobler I. (1992). Behavioral sleep in the Asian elephant in captivity. *Sleep* 15 1–12. 10.1093/sleep/15.1.1 1557589

[B143] Tobler I. (2011). “Phylogeny of sleep regulation,” in *Principles and Practice of Sleep Medicine*, 5th Edn, eds Kryger M. H. Roth T. Dement W. C. (Amsterdam: Elsevier Health Sciences), 112–125.

[B144] Tobler I. Borbély A. A. (1988). Sleep and EEG spectra in the pigeon (*Columba livia*) under baseline conditions and after sleep deprivation. *J. Comp. Physiol. A* 163 729–738. 10.1007/BF00604050

[B145] Tobler I. Schwierin B. (1996). Behavioural sleep in the giraffe (*Giraffa camelopardalis*) in a zoological garden. *J. Sleep Res.* 5 21–32. 10.1046/j.1365-2869.1996.00010.x 8795798

[B146] Trites A. W. Lestenkof P. Battaile B. (2009). *Identifying Foraging Habitat of Lactating Northern Fur Seals and the Spatial Overlap with Commercial Fisheries in the Eastern Bering Sea. NPRB Project* 636 Final Report. Vancouver: North Pacific Universities Marine Mammal Research Consortium.

[B147] Valencia Garcia S. Brischoux F. Clément O. Libourel P. A. Arthaud S. Lazarus M. (2018). Ventromedial medulla inhibitory neuron inactivation induces REM sleep without atonia and REM sleep behavior disorder. *Nat. Commun.* 9:504. 10.1038/s41467-017-02761-0 29402935 PMC5799338

[B148] van der Meij J. Martinez-Gonzalez D. Beckers G. J. L. Rattenborg N. C. (2019a). Intra-“cortical” activity during avian non-REM and REM sleep: variant and invariant traits between birds and mammals. *Sleep* 42:zsy230. 10.1093/sleep/zsy230 30462347

[B149] van der Meij J. Martinez-Gonzalez D. Beckers G. J. L. Rattenborg N. C. (2019b). Neurophysiology of avian sleep: comparing natural sleep and isoflurane anesthesia. *Front. Neurosci.* 13:262. 10.3389/fnins.2019.00262 30983954 PMC6447711

[B150] van Twyver H. Allison T. (1972). A polygraphic and behavioral study of sleep in the pigeon (*Columba livia*). *Exp. Neurol.* 35 138–153. 10.1016/0014-4886(72)90065-9 5026409

[B151] Villablanca J. (1966). Behavioral and polygraphic study of ”sleep” and ”wakefulness” in chronic decerebrate cats. *Electroencephalogr. Clin. Neurophysiol.* 21 562–577. 10.1016/0013-4694(66)90175-1 4162886

[B152] Vyazovskiy V. V. Borbély A. A. Tobler I. (2000). Unilateral vibrissae stimulation during waking induces interhemispheric EEG asymmetry during subsequent sleep in the rat. *J. Sleep Res.* 9 367–371. 10.1046/j.1365-2869.2000.00230.x 11123523

[B153] Vyazovskiy V. V. Cirelli C. Tononi G. (2011a). Electrophysiological correlates of sleep homeostasis in freely behaving rats. *Prog. Brain Res.* 193 17–38. 10.1016/B978-0-444-53839-0.00002-8 21854953 PMC3160719

[B154] Vyazovskiy V. V. Olcese U. Hanlon E. C. Nir Y. Cirelli C. Tononi G. (2011b). Local sleep in awake rats. *Nature* 472 443–447. 10.1038/nature10009 21525926 PMC3085007

[B155] Vyazovskiy V. V. Tobler I. (2008). Handedness leads to interhemispheric EEG asymmetry during sleep in the rat. *J. Neurophysiol.* 99 969–975. 10.1152/jn.01154.2007 18077659

[B156] Walker J. M. Berger R. J. (1972). Sleep in the domestic pigeon (*Columba livia*). *Behav. Biol.* 7 195–203. 10.1016/S0091-6773(72)80199-8 4339457

[B157] Warren W. C. Hillier L. W. Marshall Graves J. A. Birney E. Ponting C. P. Grützner F. (2008). Genome analysis of the platypus reveals unique signatures of evolution. *Nature* 453 175–183. 10.1038/nature06936 18464734 PMC2803040

[B158] Watanabe M. Ito H. Masai H. (1983). Cytoarchitecture and visual receptive neurons in the Wulst of the Japanese quail (*Coturnix coturnix japonica*). *J. Comp. Neurol.* 213 188–198. 10.1002/cne.902130206 6302136

[B159] Weimerskirch H. Bishop C. Jeanniard du Dot T. Prudor A. Sachs G. (2016). Frigate birds track atmospheric conditions over months-long transoceanic flights. *Science* 353 74–78. 10.1126/science.aaf4374 27365448

[B160] Wester J. C. Contreras D. (2012). Columnar interactions determine horizontal propagation of recurrent network activity in neocortex. *J. Neurosci.* 32 5454–5471. 10.1523/JNEUROSCI.5006-11.2012 22514308 PMC3415278

[B161] Yang Y. Cao P. Yang Y. Wang S. R. (2008). Corollary discharge circuits for saccadic modulation of the pigeon visual system. *Nat. Neurosci.* 11 595–602. 10.1038/nn.2107 18391942

[B162] Yasuda T. Yasuda K. Brown R. A. Krueger J. M. (2005). State-dependent effects of light-dark cycle on somatosensory and visual cortex EEG in rats. *Am. J. Physiol. Regul. Integr. Comp. Physiol.* 289 R1083–R1089. 10.1152/ajpregu.00112.2005 16183627

[B163] Zhao X. Sun H. Tang Z. Flanders J. Zhang S. Ma Y. (2010). Characterization of the sleep architecture in two species of fruit bat. *Behav. Brain Res.* 208 497–501. 10.1016/j.bbr.2009.12.027 20043956

